# Pre-treatment bronchoscopic evaluation in a case of relapsing polychondrits

**DOI:** 10.1186/s12890-023-02400-z

**Published:** 2023-04-03

**Authors:** Kosumi Kumagai, Hajime Tsuruoka, Kei Morikawa, Hiroshi Handa, Masamichi Mineshita

**Affiliations:** Division of Respiratory Medicine, Department of Internal Medicine, St. Marianna University School of Medicine, Kawasaki, 216-8511 Japan

**Keywords:** Bronchoscopic intervention, Bronchomalacia, Relapsing polychondrits

## Abstract

**Background:**

Relapsing polychondritis (RP) is a chronic and recurrent inflammatory disease of the cartilage tissues in the body. The cause of RP is unknown, and since it is a rare disease with symptoms that affect multiple organs, diagnosis is often delayed.

**Case presentation:**

A 62-year-old woman with no smoking history visited our institution complaining of fever, cough, and dyspnoea. Chest CT showed a stenosis from the left main bronchus to the left lower lobe branch. Bronchoscopy visualised intense erythema and oedema at the left main bronchus, with airway narrowing. Biopsy of the ear revealed degenerative vitreous cartilage and fibrous connective tissue with a mild inflammatory cell infiltrate. She was subsequently diagnosed with RP and administered systemic corticosteroid therapy. Her symptoms improved rapidly, and post-treatment bronchoscopy revealed that although mild erythema of the airway epithelium remained, oedema markedly improved, and the airway stenosis was resolved.

**Conclusions:**

We report a case where pre-treatment bronchoscopy was able to visually confirm RP at the acute stage. Since RP is difficult to diagnose, severe airway narrowing can occur prior to diagnosis. Therefore, to determine the stage of the disease, it is helpful to perform bronchoscopic observation before treatment. However, bronchoscopic observation before treatment should be performed by experienced bronchoscopists due to the risk of airway obstruction.

## Background

Relapsing polychondritis (RP) is characterized by repeated inflammation of cartilages throughout the body, which can cause delays in diagnosis [1]. Among the symptoms observed, auricular chondritis is the most common (78% of cases), inflammation of the airway cartilage (50%), nasal cartilage (39%), and articular cartilage (39%) [2]. Bronchoscopy in patients with advanced airway stenosis can cause airway obstruction and not all patients can be examined [3]. We herein report a rare case of RP where the bronchial lumen could be observed by bronchoscope at pre- and post-treatment, giving valuable insight to the affected airway and the post-therapeutic response.

## Case presentation

A 62-year-old woman with no smoking history experienced fever and back pain for five months before visiting our hospital. One week prior to her visit, she complained of fever, cough, and dyspnoea. Her vital signs at admission were normal, except for a slight fever and high blood pressure. Wheezing was heard on physical examination, and she had pain in the nasal area.

**Fig. 1:**
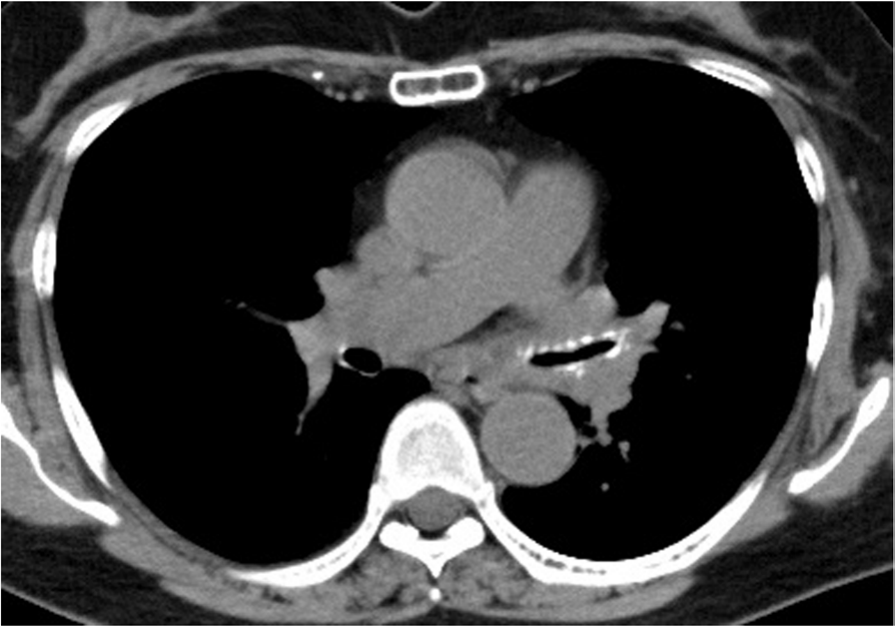
Chest CT imaging on admission showed a marked stenosis from the left main bronchus to the inferior lobe with suspected degeneration of cartilage

**Fig. 2:**
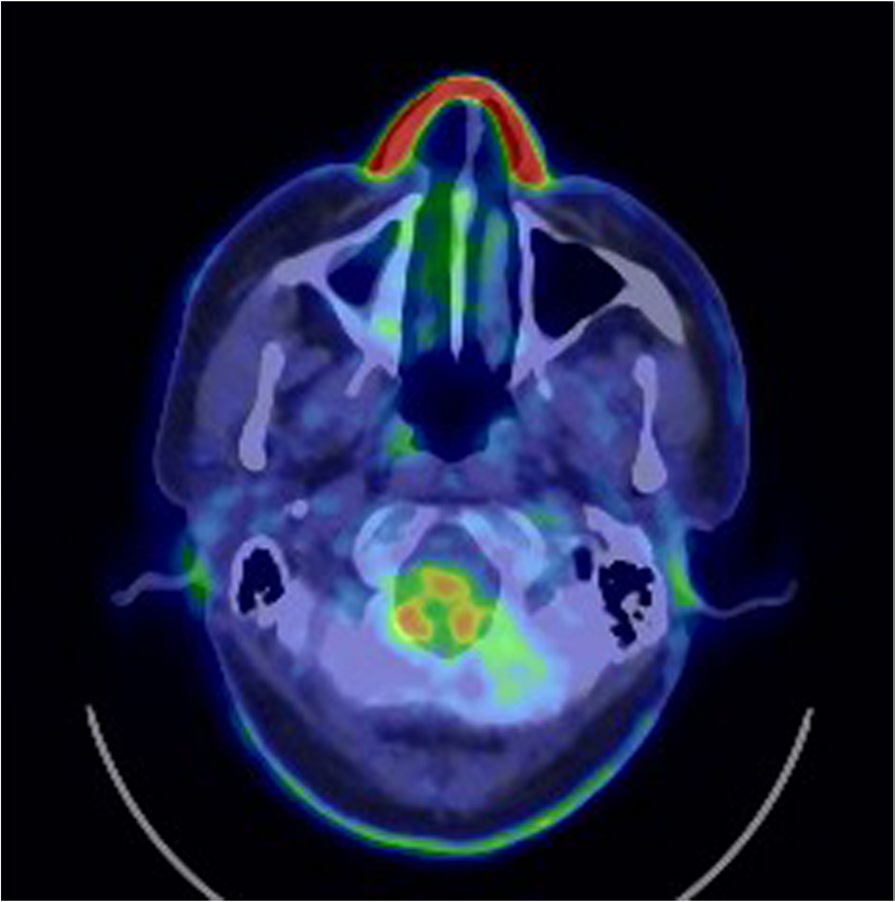
PET-CT taken 3 weeks before admission showed FDG accumulation in the nasal root

A blood test revealed an elevated inflammatory response, with a white blood cell count of 112,000/μL and CRP of 17.89 mg/dL. There was no apparent hepatic dysfunction, electrolyte abnormalities or auto immune antibodies. Pulmonary function tests revealed a forced expiratory volume in 1 s (FEV1) of 2.08 L, forced vital capacity (FVC) of 2.66 L, FEV1/FVC of 78.02%, and a FEV1% predicted of 99.6%. Chest CT showed a stenosis from the left main bronchus to the left lower lobe branch (Fig. 1). PET-CT images taken at the previous hospital showed high FDG accumulation in the nose (Fig. 2).

**Fig. 3:**
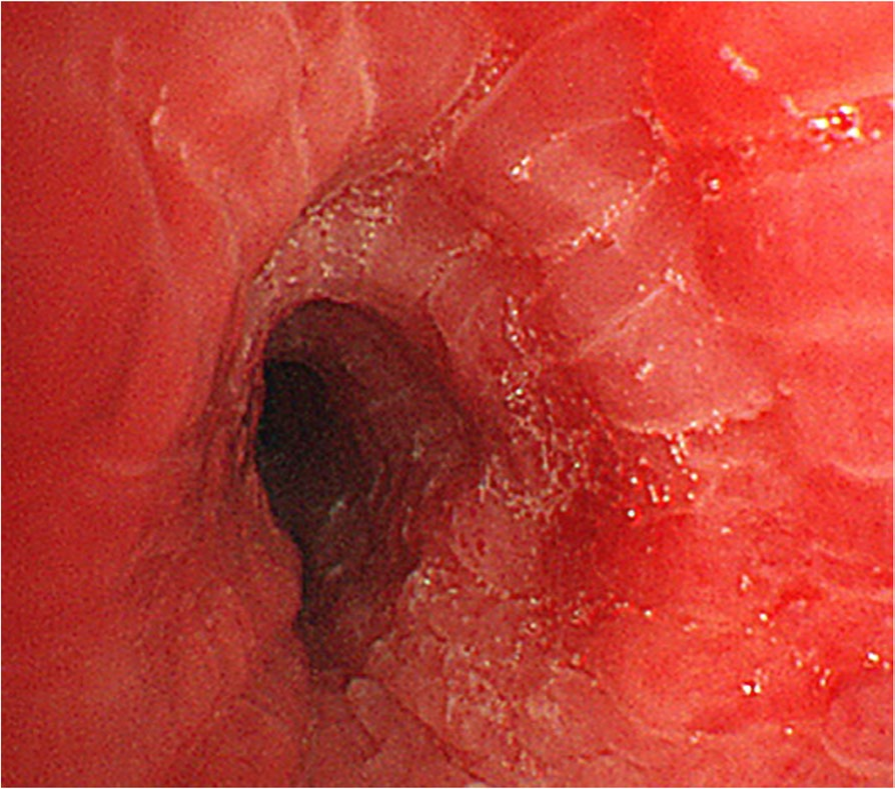
The left main bronchus and lower lobar branch showed severe erythema and swelling, making insertion of the bronchoscope difficult

**Fig. 4:**
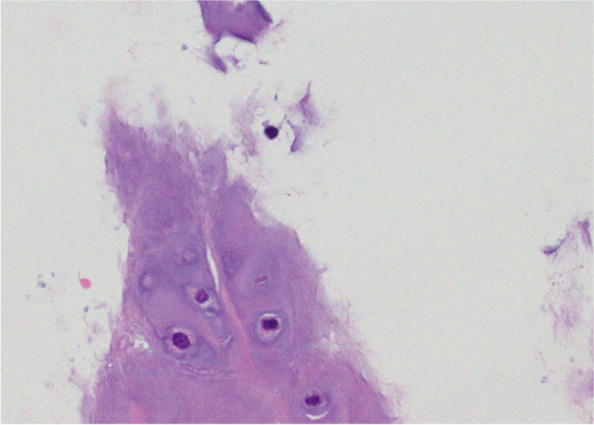
Histological specimen taken from the right auricular cartilage showed a small amount of lymphocytic infiltrate in the vitreous cartilage

The patient was urgently admitted to hospital. Bronchoscopy was performed under SpO2 monitoring and oxygenation. Pharyngeal anaesthesia was administered with xylocaine (5 mL). Xylocaine was delivered through the bronchoscope to the vocal cords, the main bronchial tube, and entire tracheobronchial tree. Sedatives were not used to prevent airway collapse. Bronchoscopy performed on the fourth day showed a normal epithelium from the vocal cords to the tracheal bifurcation. However, intense erythema and oedema were observed at the left main bronchus, and the airway had narrowed (Fig. 3). A biopsy of the right auricular cartilage was performed on the same day and revealed degenerative vitreous cartilage and fibrous connective tissue with mild inflammatory cell infiltrate (Fig. 4). According to McAdam's diagnostic criteria, the following four categories were satisfied for a diagnosis of RP: bilateral auricular chondritis, nasal chondritis, tracheal chondritis, and biopsy pathology findings consistent with chondritis.

**Fig. 5:**
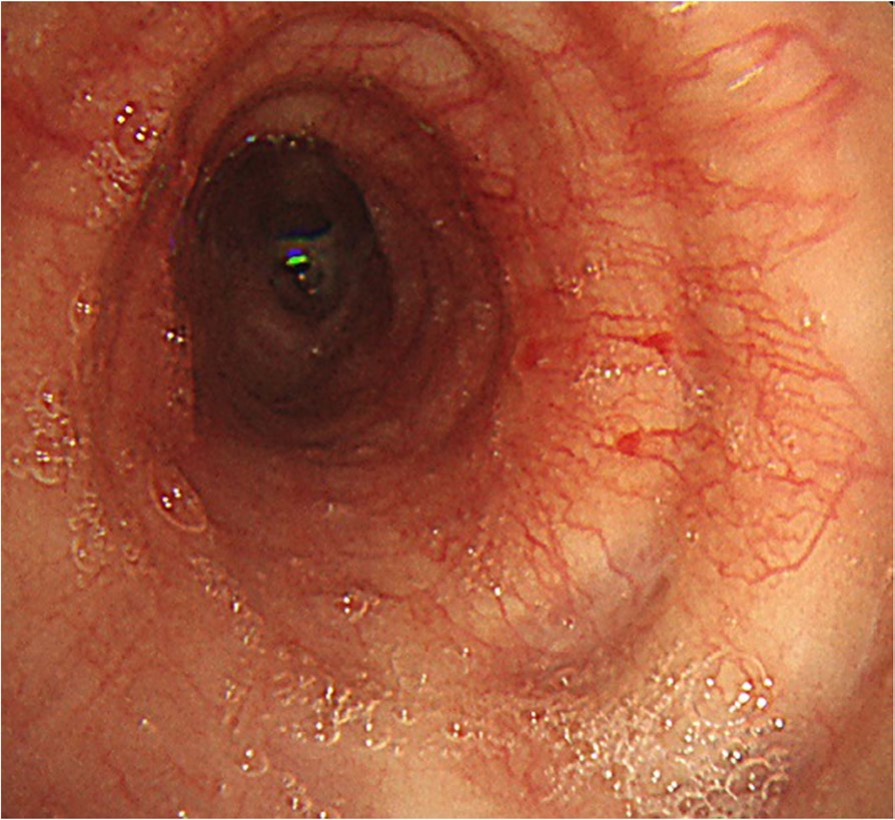
Bronchoscopy on day 12 showed that the erythema and swelling of the left inferior lobe had improved markedly

Methylprednisolone 1000 mg/day for three days was administered from day 4, and her dyspnoea rapidly improved. On day 5, the patient was administered prednisolone 60 mg/day which was tapered to 50 mg/day on day 19, 40 mg/day on day 26, and 30 mg/day on day 31. On the 12th day, a second bronchoscopy was performed and although mild erythema of the airway epithelium was observed, oedema markedly improved, and the airway stenosis was resolved (Fig. 5).

**Fig. 6:**
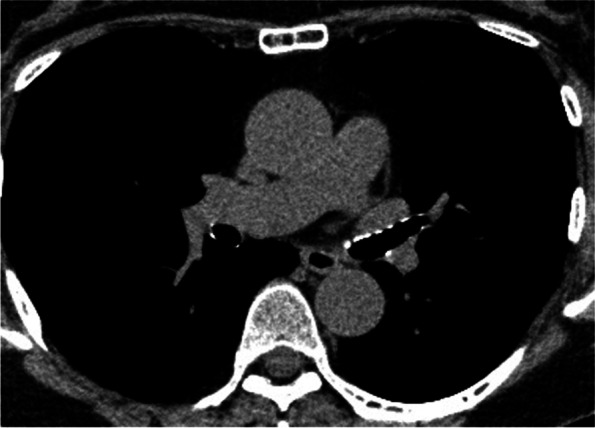
Chest CT imaging showed the stenosis markedly improved

Cyclosporine 150 mg/day was started on day 17, and the dose was increased to 200 mg/day on day 23. On the 29th day, CT imaging showed remarkable a improvement for the airway stenosis (Fig. 6). On day 31, the patient was discharged from our institution.

## Discussion and conclusions

CT is one of the most minimally invasive evaluations for airway lesions [4]. However, neither dynamic CT nor virtual bronchoscopy can evaluate inflammatory findings and the softening of the airway epithelium in real-time. Conversely, bronchoscopy is able to diagnose tracheobronchomalacia because it can capture dynamic airway properties [5]. In this case, CT imaging showed a stenosis with possible cartilage degeneration and subsequent pre-treatment bronchoscopy was performed to visually confirm the stenosis and evaluate the epithelial changes in the airway.

The progression of airway lesions with RP is described as follows: swelling due to inflammation of the airway epithelium in the acute stage, a scar-like narrowing due to fibrosis in the chronic stage, and the eventual destruction of tracheal cartilage, leading to irreversible respiratory failure [6]. Since airway malacia, classified as sabre sheath and circumferential type [7], results in irreversible changes the early detection and diagnosis of RP is important. In this case, pre-treatment bronchoscopic findings revealed a circumferential airway with inflammation and oedema at the trachea which was identified as acute stage. Early steroid treatment was administered resulting in an improvement in airway inflammation, which was confirmed by post-treatment bronchoscopy.

The McAdam's criteria requires meeting three of the following conditions: bilateral auricular chondritis, nonerosive seronegative inflammatory arthritis, nasal chondritis, ocular inflammation, respiratory tract chondritis, and audio vestibular damage [8]. To determine the stage of the disease, it is helpful to perform bronchoscopic observation before treatment. Ernst et al. reported that when bronchoscopy was performed in 23 of 31 RP patients, typical bronchoscopic findings were airway malacia and subglottic stenosis [9]. In the present case, our patient complained of cough, dyspnoea, and nasal pain prior to visiting our hospital. Since pulmonary function tests revealed there was no obstruction or risk of respiratory failure, bronchoscopy was performed to evaluate the bronchial lesion prior to steroid therapy. After visual evaluation by bronchoscope, airway biopsy was judged to be high risk and steroid therapy was administered.

The early introduction of steroids prevented the development of airway epithelium fibrosis and possible bronchomalacia. However, even with the early introduction of steroids and immunosuppressive agents, approximately 40% of patients with airway involvement require airway intervention [10]. These interventions can include tracheotomy when the stenosis is confined to the larynx and the upper airway obstruction worsens, or NIPPV and silicone stenting if tracheomalacia or bronchomalacia have already developed [11].

In this case, bronchoscopy might not have been necessary for a definitive diagnosis, but we believe that bronchoscopy was important to differentiate tumour lesions and identify the progression of the lesion. Although bronchoscopy can be informative for cases suspected of RP, it cannot be easily performed in all patients and therefore, should be performed at a skilled facility due to the risk of airway obstruction.

## Data Availability

The datasets used and/or analysed during the current study are available from the corresponding author on reasonable request.

## Abbreviations

FDG: Fluorodeoxyglucose
NIPPV: Non-invasive positive pressure ventilation
PET-CT: Positron emission tomography-computed tomography
RP: Relapsing polychondritis
